# *Torosaurus* Is Not *Triceratops*: Ontogeny in Chasmosaurine Ceratopsids as a Case Study in Dinosaur Taxonomy

**DOI:** 10.1371/journal.pone.0032623

**Published:** 2012-02-29

**Authors:** Nicholas R. Longrich, Daniel J. Field

**Affiliations:** Department of Geology and Geophysics, Yale University, New Haven, Connecticut, United States of America; University of Maryland, United States of America

## Abstract

**Background:**

In horned dinosaurs, taxonomy is complicated by the fact that the cranial ornament that distinguishes species changes with age. Based on this observation, it has been proposed that the genera *Triceratops* and *Torosaurus* are in fact synonymous, with specimens identified as *Torosaurus* representing the adult form of *Triceratops*. The hypothesis of synonymy makes three testable predictions: 1) the species in question should have similar geographic and stratigraphic distributions, 2) specimens assigned to *Torosaurus* should be more mature than those assigned to *Triceratops*, and 3) intermediates should exist that combine features of *Triceratops* and *Torosaurus*. The first condition appears to be met, but it remains unclear whether the other predictions are borne out by the fossil evidence.

**Methodology/Principal Findings:**

We assessed the relative maturity of *Torosaurus* and *Triceratops* specimens by coding skulls for characters that vary with maturity, and then using a clustering analysis to arrange them into a growth series. We found that a well-defined sequence of changes exists in horned dinosaurs: development of cranial ornament occurs in juveniles, followed by fusion of the skull roof in subadults, and finally, the epoccipitals, epijugals, and rostral fuse to the skull in adults. Using this scheme, we identified mature and immature individuals of both *Torosaurus* and *Triceratops*. Furthermore, we describe the ventral depressions on the frill of *Triceratops*, and show that they differ in shape and position from the parietal fenestrae of *Torosaurus*. Thus, we conclude that these structures are not intermediates between the solid frill of *Triceratops* and the fenestrated frill of *Torosaurus*.

**Conclusions/Significance:**

*Torosaurus* is a distinct genus of horned dinosaur, not the adult of *Triceratops*. Our method provides a framework for assessing the hypothesis of synonymy through ontogeny in the fossil record.

## Introduction

Understanding the diversity and relationships of ancient life requires first assigning fossils to species. Classifying fossils is fundamental to paleontology [1], [2], but in practice it can present major challenges. Before one can use variation to classify species, it is necessary to understand the nature of that variation. That is, do the differences between two fossils represent variation between different species, which is a result of separate evolutionary histories, or do these differences reflect variation within a single species, which can result from variation within a population, sexual dimorphism, or change in morphology over the course of development?

The horned dinosaurs, or Ceratopsidae, vividly illustrate the difficulties of separating within-species variation from between-species variation. At the end of the 19th century, fossils of giant horned dinosaurs were discovered in the uppermost Cretaceous (upper Maastrichtian) of the American West [3], [4], [5], [6], [7], and over the next century, ceratopsids were discovered in upper Maastrichtian rocks across the western United States and Canada [8]. No two specimens are entirely identical, and as a result, a remarkable number of names have been attached to these fossils by paleontologists, with no fewer than ten genera and 22 species being formally erected over the past century and a half [4], [5], [6], [9], [10], [11], [12], [13], [14], [15], [16], [17], [18], [19], [20], [21], [22].

**Figure 1:**
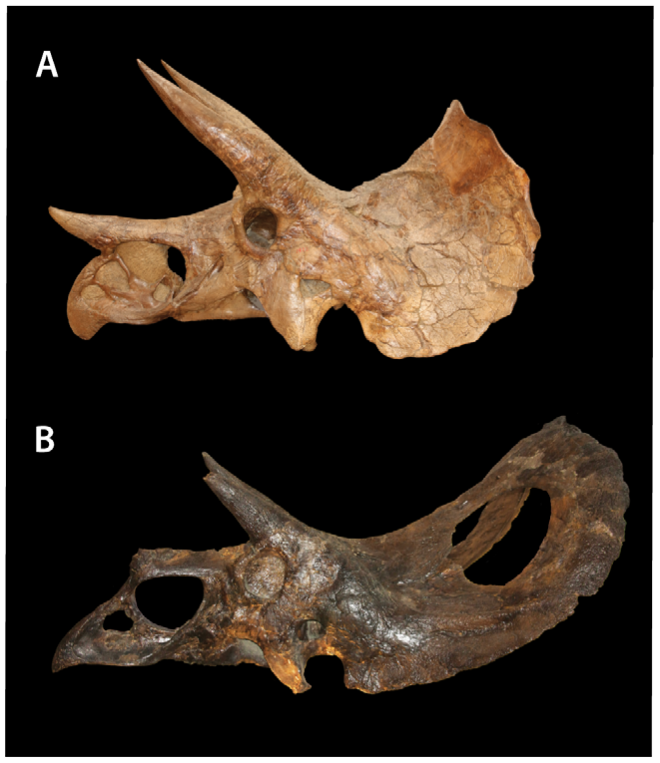
*Torosaurus* and *Triceratops* compared. A, *Triceratops prorsus* YPM 1822 and B, *Torosaurus latus* ANSP 15192. *Triceratops* is characterized by a short frill with a flat squamosal, an upturned caudal margin of the frill, the absence of fenestrae, and a midline epiparietal. *Torosaurus* is characterized by an elongate frill with a straighter edge, a concave squamosal, and lack of upturning of the frill.

In recent years, however, paleontologists have become far more conservative in naming and recognizing species [1], [2]. As a result, most of the genera and species erected for late Maastrichtian horned dinosaurs are now considered invalid, either because they were named on the basis of poor fossil material, or because the type fossils are thought to lie within the range of variation of existing species [23], [24], [25], [26], [27], [28]. However, it is generally agreed that the late Maastrichtian horned dinosaurs represent at least two distinct genera [23], [25], [26], [29], [30]: *Triceratops*, characterized by a short, solid frill, and *Torosaurus*, characterized by a long, open frill (Figure 1). *Triceratops* is in turn divided into two species: *T*. *horridus*, distinguished by a short nose horn and long rostrum, and *T*. *prorsus*, characterized by a long nose horn and short rostrum [25], [31].

However, it has become apparent that some dinosaurs underwent dramatic morphological changes as they matured [32], [33], [34], [35], [36], [37], [38]. In particular, the frills and horns that are so critical to understanding the taxonomy of ceratopsids [24], [30], [39] have been shown to change markedly over the course of development, with elements variously becoming elaborated, reduced, or fused as the animals matured [32], [33], [34], [35]. In light of this fact, it has been proposed that the differences between specimens assigned to *Triceratops* and *Torosaurus* could actually reflect differences in maturity, with specimens assigned to *Torosaurus* simply representing the adult morphology of *Triceratops* [31], [40], [41], [42]. This idea is controversial [26], [43], but if corroborated, it would have significant implications for understanding the diversity of dinosaurs, because it would mean that the differences now used to recognize many ceratopsid species could simply result from changes that occurred as the animals grew. In light of this controversy, we examine the synonymy of *Torosaurus* and *Triceratops* as a case study in dinosaur taxonomy.

The hypothesis that *Torosaurus* and *Triceratops* are growth stages of a single genus makes three testable predictions about these fossils that are necessary, but by themselves insufficient, to infer synonymy. If *Torosaurus* and *Triceratops* are different growth stages of a single animal, then the two forms must 1) have similar distributions in the fossil record, 2) differ in their relative maturity, and 3) be linked by morphological intermediates. All three of these predictions must be unambiguously satisfied for the hypothesis of synonymy to be supported.

### 1. Distribution in the Fossil Record

**Figure 2:**
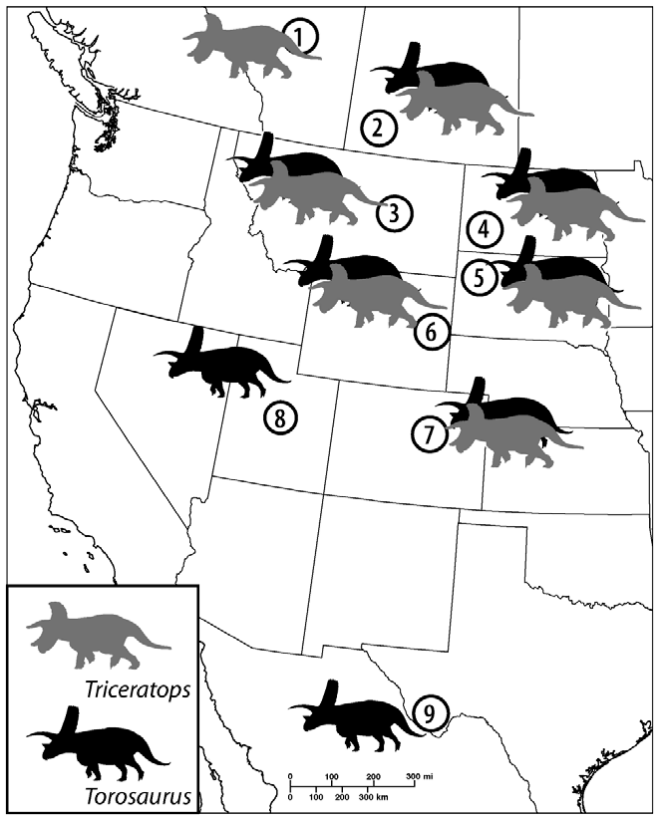
Distribution of *Torosaurus* and *Triceratops*. 1, Scollard Formation, Alberta; 2, Frenchman Formation, Saskatchewan, 3, Hell Creek Formation, Montana; 4, Hell Creek Formation, North Dakota; 5, Hell Creek Formation, South Dakota; 6, Lance Formation, Wyoming; 7, Denver Formation, Colorado; 8, North Horn Formation, Utah; 9, Javelina Formation, Texas.

If *Torosaurus* and *Triceratops* represent a single dinosaur, then the two forms must have lived at the same time, and should have similar, if not identical, geographic ranges. This prediction appears to be met. Both *Torosaurus* and *Triceratops* are known exclusively from the late Maastrichtian of western North America [8]. Figure 2 shows the distribution of the two forms across North America; data are from a recent review of dinosaur distributions [8] with two edits: *Torosaurus* is added to the Denver Formation of Colorado [44] and removed from the fauna of the Scollard Formation (we were unable to locate any published references or fossils supporting its occurrence in Alberta).

The ranges of *Torosaurus* and *Triceratops* therefore overlap from as far north as Saskatchewan to as far south as Colorado. Only in the extreme north and south do the two fail to overlap: *Triceratops*, but not *Torosaurus*, is known from Alberta, while *Torosaurus*, but not *Triceratops*, is known from the American Southwest. However, very few skeletons are known from these localities, so the lack of overlap in these formations could easily represent a sampling artifact. *Torosaurus* and *Triceratops* therefore have similar distributions in the fossil record, consistent with synonymy.

### 2. Relative age of *Torosaurus* and *Triceratops*

Synonymy predicts that the different morphologies are associated with different growth stages; that is, if *Torosaurus* represents the adult form of *Triceratops*, then all individuals of *Torosaurus* must be mature, and all individuals of *Triceratops* must be relatively immature. Scannella and Horner sought to test this prediction by examining the osteohistology of the postorbital horns [31] while Horner and Lamm studied the histology of the parietal [42]. The *Torosaurus* individual examined in the first study was found to have undergone more bone remodeling than the *Triceratops* specimens that were analyzed, which was taken as evidence that the animal was more mature. However, only a single specimen of *Torosaurus* was sampled in either study, making it impossible to determine whether this pattern holds for *Torosaurus* and *Triceratops* in general [43]; neither is it clear that the degree of remodeling can reliably be used to infer ontogenetic stage in living animals. Furthermore, there is evidence that one specimen of *Torosaurus*, YPM 1831, represents an immature animal, because some of its cranial elements appear to be unfused [43]. However, this suggestion has not been confirmed. Thus, it remains unclear whether *Torosaurus* consistently differs from *Triceratops* in terms of maturity.

**Figure 3:**
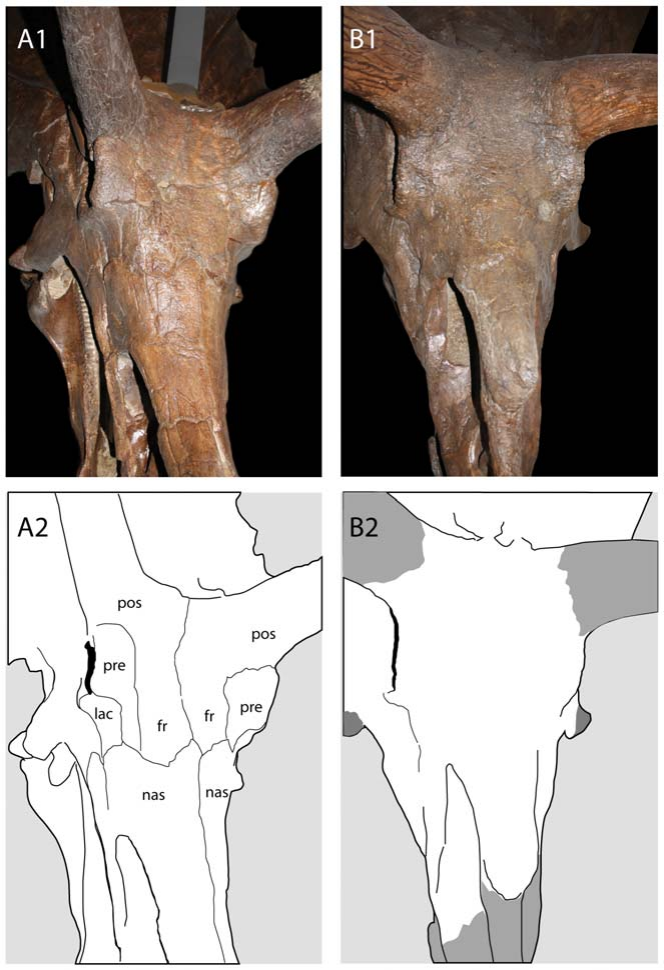
Fusion of the skull roof in Chasmosaurinae. A, *Triceratops prorsus* YPM 1823; B, *Torosaurus latus* YPM 1830. Abbreviations: fr, frontal; lac, lacrimal; nas, nasal; pos, postorbital; pre, prefrontal.

**Figure 4:**
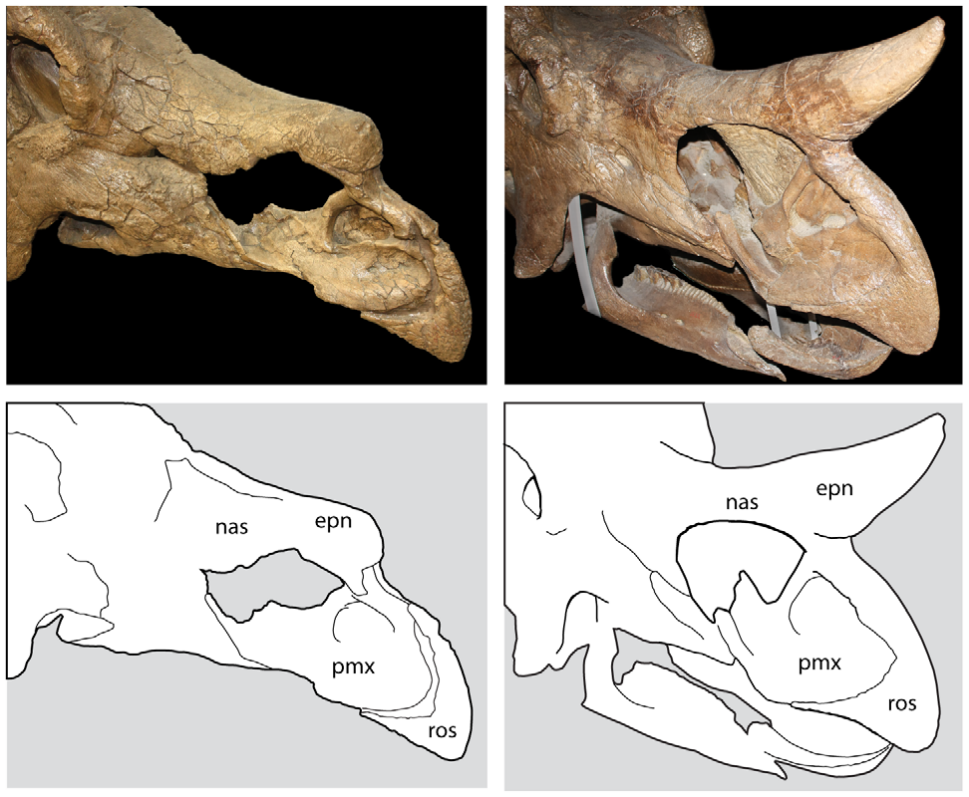
Fusion of the rostrum in Chasmosaurinae. **A**, *Triceratops horridus* USNM 1201; B, *Triceratops prorsus* YPM 1822. Abbreviations: epn, epinasal; nas, nasal; pmx, premaxilla; ros, rostral.

Ideally, maturity would be inferred by histological studies of long bones, as previously done for a number of dinosaur species [45], [46], [47], [48], [49]; however, most *Torosaurus* skulls lack associated skeletons. Nonetheless, it should be possible to infer relative age in *Torosaurus* by examining morphological changes that occur in the skull as an animal matures. Many changes in skull shape occur as horned dinosaurs grow: the frill becomes elongate, the postorbital horns become long, massive, and procumbent [34], and the rostrum becomes deeper. The surface texture of the bones of the face and frill is also modified, changing from a striated texture, which characterizes young, rapidly growing bone [50], to a texture that is gnarled and rugose, with large canals for blood vessels [51]. Skull bones also fuse in mature horned dinosaurs [33], [34], [35], [52]; this includes fusion of the frontals, nasals, and circumorbital bones to form a single unit (Figure 3) [33], fusion of the rostral to the premaxillae, the premaxillae to the nasals (Figure 4), and fusion of the exoccipitals and basioccipitals. Dermal ossifications, including the epiparietals, episquamosals, epijugals, and epinasal, also fuse to underlying skull bones [34], [35]. In extant mammals, cranial fusions tend to occur in a distinct sequence [53], [54], [55]. Skull fusion in mammals does not indicate cessation of growth [53], but increase in size following suture closure is limited [53] because bone can no longer be deposited between the sutures. Similarly, skull fusion in lizards generally occurs late in life, either as the animal nears full size, or after the animal reaches maturity [56]. Thus, closure of cranial sutures appears to reflect a reduction in growth rates, and can be assumed to reflect the attainment of skeletal maturity. It follows that, as done previously for *Triceratops* [52], skull sutures can be used to infer the relative age of skulls assigned to *Torosaurus*.

Previous studies of development have created growth series by using ontogenetically variable characters to arrange specimens into a series, using parsimony-based clustering analyses [37], [57], [58]. We employed this method for *Torosaurus* and *Triceratops* skulls, coding fossils assigned to these genera for ontogenetically variable characters, and then conducting a clustering analysis to create a developmental sequence.

### 3. Morphological intermediates

If *Torosaurus* developed from *Triceratops* through a series of gradual morphological changes, then intermediate forms must exist. However, *Triceratops* and *Torosaurus* exhibit major differences that are difficult to reconcile with the hypothesis of synonymy. One of these is the differing number of parietal epoccipitals on the frill of each animal [43]; *Triceratops* has 5–7, whereas *Torosaurus* has 10 or more. The most conspicuous difference, however, concerns the fenestrae of the *Torosaurus* frill. The parietal of *Torosaurus* exhibits a pair of large, circular openings, whereas the parietal of *Triceratops* is a solid sheet of bone. If the *Torosaurus* morph did develop from *Triceratops*, then forms exhibiting the incipient development of parietal fenestrae should exist. Scannella and Horner suggest that such intermediates are known. They described depressions on the ventral surface of the parietal of *Triceratops* as incipient parietal fenestrae [31], and proposed that the skull of USNM 2412 (“*Nedoceratops hatcheri*”) — a *Triceratops*-like skull with an opening in the parietal — represents an intermediate between *Triceratops* and *Torosaurus* [41]. Below, we assess these putative intermediate morphologies, paying particular attention to the structure of the ventral fossae in *Triceratops* and comparing them to the parietal openings of *Torosaurus*.

### Institutional Abbreviations

AMNH, American Museum of Natural History, New York, New York; ANSP, Academy of Natural Sciences of Philadelphia, Philadelphia, Pennsylvania; BHI, Black Hills Institute of Geological Research, Hill City, South Dakota; BSP, Bayeriche Staatsammlung für Paläontologie und Geologie, Munich, Germany; CMN, Carnegie Museum of Natural History, Pittsburgh, Pennsylvania; GMNH, Gunma Museum of Natural History, Gunma, Japan; MNHN Muséum National d'Histoire Naturelle, Paris, France; MOR, Museum of the Rockies, Bozeman, Montana; MPM, Milwaukee Public Museum, Milwaukee, Wisconsin; OMNH, Oklahoma Museum of Natural History, Norman, Oklahoma; SMNH, Saskatchewan Museum of Natural History, Regina and Eastend, Saskatchewan; UCMP, University of California Museum of Paleontology, Berkeley, California; USNM, United States National Museum, Washington, DC; UW, University of Wyoming, Laramie, Wyoming; YPM, Yale Peabody Museum, New Haven, Connecticut.

## Materials and Methods

We coded fossils referred to *Triceratops* and *Torosaurus* for 24 characters (Table S1) that describe the development of cranial ornament, bone surface texture, fusion between skull bones, and fusion of dermal ossifications to the skull. When possible, observations were made directly from the fossils; otherwise, coding was done from the literature (see Supporting Information S1). Cranial elements were coded as fused when the sutures between elements were obliterated externally. Peripheral ossifications including the epoccipitals, epijugal, and epinasal were coded as fused where a bony connection had developed between the ossification and the underlying skull bone. The rostral is coded as fused when the suture separating the dorsal part of the rostral and the premaxillae was eliminated. Data are shown in Table S2 (see also Supporting Information S1).

Specimens are referred to *Torosaurus* on the basis of the following characters: parietal fenestrate, 10 or more epiparietals, squamosals with a concave dorsal surface delimited by the thickened inner edge of the squamosal (medial bar), squamosals with a straight lateral edge [26], [29], [43]. Specimens are referred to *Triceratops* on the basis of the following derived characters: parietals lacking fenestrae, posterior border of parietal upturned, five to seven epiparietals, posterior blade of squamosal flat and lacking medial bar. *Triceratops prorsus* exhibits three additional derived characters that distinguish it from *Triceratops horridus* and *Torosaurus*: squamosal with a strongly convex lateral margin, nasal process of premaxilla vertically oriented, elongate nasal horn [25], [26], [29].

“*Nedoceratops hatcheri*” [43] matches the diagnosis of *Triceratops horridus* and is therefore treated as *T. horridus* [26]: the irregular shape of the hole in the parietal suggests that it is pathological, the small nasal horn is approached by several *Triceratops* specimens (e.g., USNM 4720; UCMP 128561) and the erect postorbital horncore [43] is not found on both sides of the animal, suggesting that it is an artifact created by crushing of the skull. *Torosaurus utahensis* [17] and *Tatankaceratops sacrisonorum* [22] were also included although the affinities of the first are problematic, and the second may represent an aberrant individual of *Triceratops prorsus* [26].“*Ojoceratops fowleri*” [20] and *Torosaurus* sp. from the Javelina Formation [21] were excluded because of the incompleteness of these specimens. A total of 36 specimens were analyzed.

To create a growth series, we used the computer program PAUP* 4.0 b10 [59] to cluster specimens using parsimony analysis. Owing to missing data and the fact that many specimens coded similarly, a very large number of shortest trees were produced. Therefore, rather than attempting to find all most parsimonious trees, we estimated the consensus by using a heuristic search algorithm to find a subset of most parsimonious trees (arbitrarily set at 250,000), and created a strict consensus tree.

## Results

### Ontogenetic Sequence Analysis

**Figure 5:**
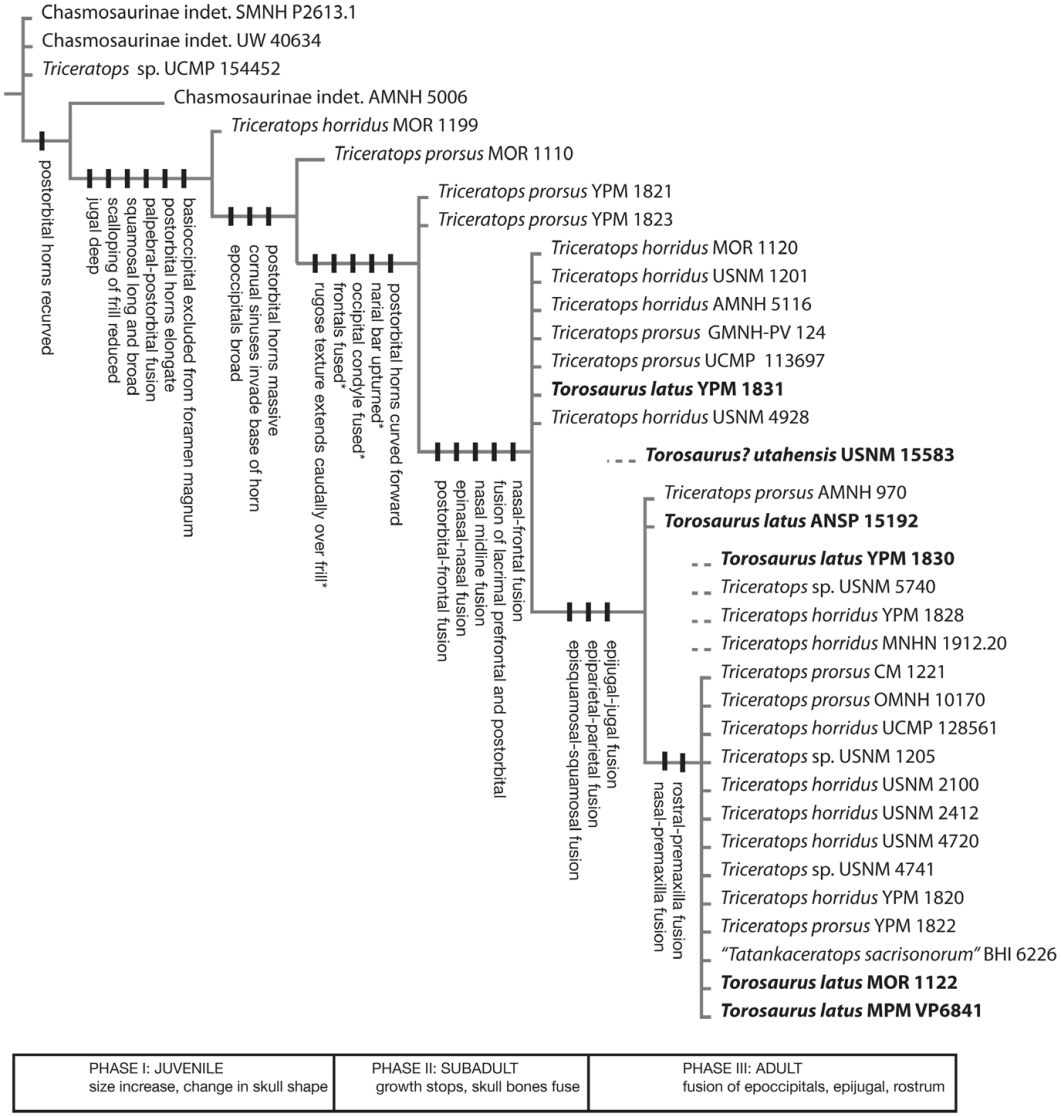
Clustering diagram with *Triceratops* and *Torosaurus* specimens arranged in developmental sequence. Inferred sequence of ontogenetic character changes is mapped onto the diagram. An initial run found that MNHN 1912.20, YPM 1830, YPM 1828 and USNM 5740 are adults and that USNM 15583 was immature. Due to missing data these specimens caused a lack of resolution and a second run of the analysis was conducted excluding these specimens. Asterisks indicate character change mappings that are provisional owing to missing or conflicting data.

Clustering analysis (Figure 5) recovers a branching diagram that corresponds to an ontogenetic series, with the specimens at the base of the tree representing juveniles, and specimens at the top representing adults. Initial runs resulted in 250,000 trees (treelength = 33, consistency index = .7879, retention index = .9381, rescaled consistency index = .7391), however, resolution was relatively poor in the strict consensus compared to the Adams consensus. Examination of the raw data revealed that five specimens were particularly problematic because they could not be coded for characters necessary to placing them precisely in the sequence. These included four specimens that clustered with adults in initial runs (*Torosaurus* YPM 1830, *Triceratops* MNHN 1912.20, *Triceratops* YPM 1828, *Triceratops* USNM 5740), and one that grouped with subadults (*Torosaurus* USNM 15583). To improve resolution, the analysis was rerun without these specimens, producing a better-resolved tree (Figure 5) (treelength = 33, consistency index = .7879, retention index = .9358, rescaled consistency index = .7373.

The reconstructed ontogenetic sequence is shown in Figure 5. Characters are mapped at the earliest node for which there is evidence of their presence. Owing to character conflict, missing data, and variability in the timing of ontogenetic changes, the sequence does not perfectly describe the development of *Triceratops* and *Torosaurus*, but rather represents an approximation of it.

The results suggest that growth in horned dinosaurs can be divided into three phases, here referred to as juvenile, subadult, and adult (Figure 5). In the first phase, the skull undergoes major changes in shape: the parietals become long and broad, frill scalloping is reduced, and the postorbital horns become long, massive, and curved forward. The second phase involves fusion of the skull, including fusion of the frontals, postorbitals, prefrontals, lacrimals, and nasals into a single element, together with the epinasal; the exoccipitals and the basioccipital also fuse to form the occipital condyle. Development of the characteristic rugose surface texture of the frill and face also begins at this time. Individuals in this second, subadult phase of development are fully as large or larger than more mature specimens, e.g. the subadult *Torosaurus* YPM 1831 has a parietal length of 132 cm, making it the largest known specimen of *Torosaurus* [29]; subadult *Triceratops* YPM 1821 and YPM 1823 measure an estimated 101 and 91 cm from the rostral to the back of the quadrates, respectively, while the mature YPM 1820 measures just 86 cm. This suggests that growth dramatically slowed in this second phase. The third, or adult, phase involves fusion of dermal ossifications, including the epiparietals, episquamosals, and epijugal to the skull. Finally, the rostral fuses to the premaxillae, which then fuse to the nasals.

Overall the sequence of changes appears to be highly conservative, but there are some exceptions. First, the development of the rugose texture of the frill appears to be ontogenetically variable, occurring earlier in some individuals than in others. In addition, *Torosaurus* YPM 1831 is unusual in having an unfused occipital condyle, which is fused in other, more immature specimens. BHI 6226, an unusually small skull described as *Tatankaceratops*, exhibits a combination of characters consistent with immaturity (lack of cornual sinuses, slender postorbital horns) and other characters consistent with adult status (e.g. fused rostral, premaxilla, epoccipitals).

**Figure 6:**
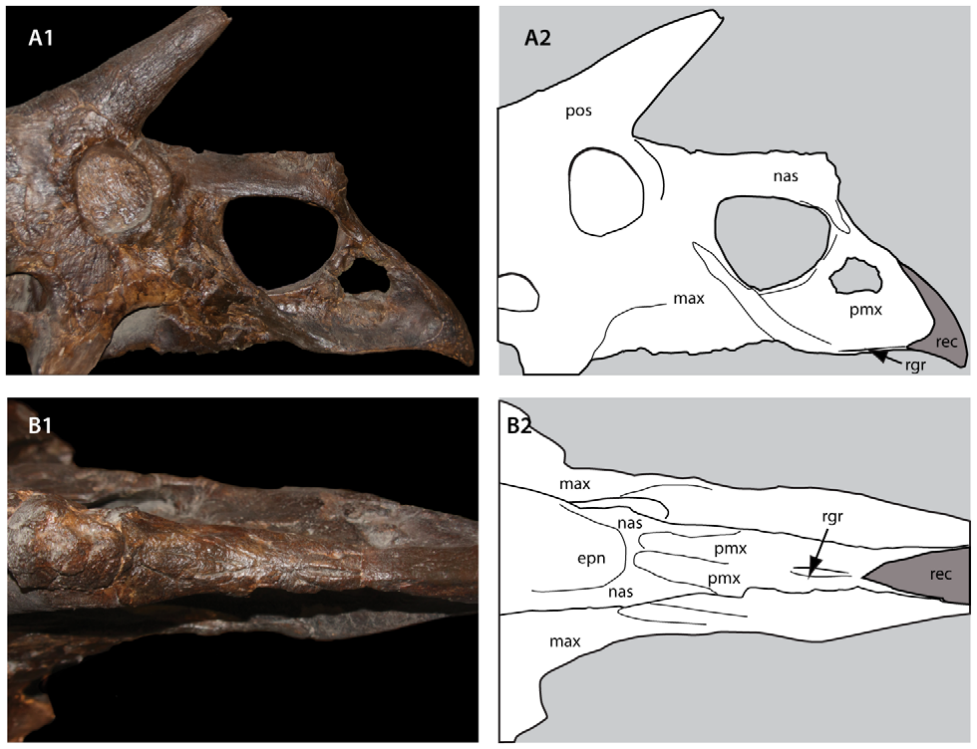
Immature features in *Torosaurus latus* ANSP 15192, a young adult. A, lateral view; B, dorsal view. Abbreviations: epn, epinasal; max, maxilla; nas, nasal; pmx, premaxilla; pos, postorbital horncore, rec, reconstruction; rgr, rostral groove.

**Figure 7:**
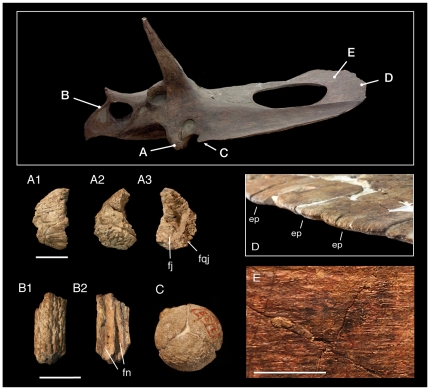
Immature features in *Torosaurus latus* YPM 1831. A, unfused epijugal. A1, anterior view, A2, lateral view; A3, medial view. B, unfused rostral. B1, dorsal view; B2, ventral view. C, occipital condyle formed of unfused exoccipitals and basioccipital. D, caudal margin of parietal showing rounded margin where unfused epoccipitals attach. E, dorsal surface of parietal showing striated surface texture. Scales = 50 mm for A and B, 20 mm for E.

Of the six specimens of *Torosaurus latus* examined here, three (MOR 1122, MPM VP P6841 and YPM 1830) were found to be adults . Whether YPM 1830 is an old adult or a young adult could not be determined because the rostral and premaxillae are missing. MOR 1122 is an old adult, as indicated by the fusion of the rostral to the premaxillae, but the presence of an open nasal-premaxilla suture [29] indicates that it is less mature than a number of *Triceratops* specimens. *Torosaurus* ANSP 15192 codes as mature for all characters except two: fusion of the premaxillae and nasals, and fusion of the rostral and premaxillae (Fig. 6). An open suture is retained between the premaxillae and the nasals (Fig. 6A); the rostral is not present (having been reconstructed), but dorsally the premaxillae bear a groove for the ventral ridge of the rostral, and the ventral margin of the premaxilla bears a groove to receive the posterior ramus. Thus the rostral appears to have fallen off prior to burial. Therefore, *Torosaurus* ANSP 15192 is a young adult. Finally, *Torosaurus* YPM 1831 exhibits a combination of mature and juvenile features, corroborating previous suggestions that the animal is immature [43]. The animal has several features suggestive of maturity: it is very large, with an elongate frill and long, massive, anteriorly oriented postorbital horns; the proximal surface of the parietal is rugose, the orbital boss is fused and, contra previous interpretations [43], the epinasal appears to be fused. However, the animal also exhibits juvenile characters. These include a free epijugal (Fig. 7A), an unfused rostral (Fig. 7B), and an unfused occipital condyle (Fig. 7C). Epoccipitals are not visible on the margin of the parietal or squamosals. Although epoccipitals can become so tightly sutured to the frill that they are difficult to identify, in *Torosaurus* YPM 1831 they instead appear to have fallen off. In *Torosaurus* YPM 1830, fused epoccipitals are identifiable by their rugose texture and distinct lateral keel; however in *Torosaurus* YPM 1831 the frill has a smooth, rounded edge (Fig. 7D). Finally, although the frill's base is rugose and bears vascular grooves, the caudal margin of the parietal has the striated surface texture (Figure 7E) associated with immature, fast-growing bone [51]. On the basis of these features, *Torosaurus* YPM 1831 represents a subadult. USNM 15583, *Torosaurus*? *utahensis*, also represents a subadult, as it has an unfused epijugal, unfused lacrimal, and unfused episquamosals. The Utah chasmosaurine has a thin frill and may have parietal fenestrae [17], suggesting that it represents *Torosaurus*. If so, USNM 15583 is another example of an immature *Torosaurus*. However the squamosal of USNM 15583 is relatively short and broad, and it is unclear whether it preserves a squamosal medial bar. Thus, referral of this species to *Torosaurus* is not certain. *Triceratops* specimens likewise range from young juveniles to very old adults with the full complement of cranial fusions (Fig. 5). A large percentage of individuals are adults, however, coding as mature for most or all characters (Table S2).

**Figure 8:**
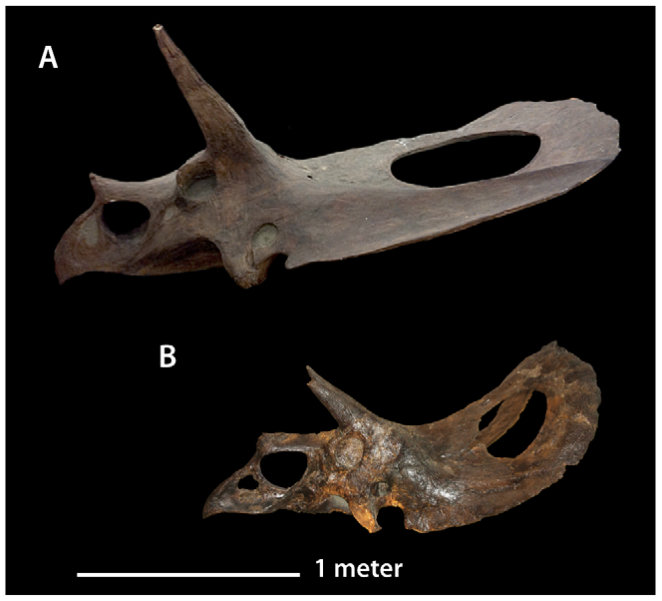
Comparison of the size of *Torosaurus latus* specimens. A, YPM 1831, a subadult, skull length >2.6 m, and B, ANSP 15192, an early adult, skull length 1.8 m. The rostrum of YPM 1831 is reconstructed and would probably have been slightly longer, as in ANSP 15192.

Surprisingly, size and maturity do not appear to be strictly correlated in *Torosaurus* (Figure 8); the ANSP skull is a young adult, yet it is roughly two-thirds the size of the giant subadult YPM 1831 (1.8 vs. 2.6 m, respectively). Similar size variation occurs in *Triceratops*: the skull of YPM 1822, an old adult, is only 1.6 m in length, whereas YPM 1828 has an estimated length of 2.4 meters.

### Morphological Intermediates

**Figure 9:**
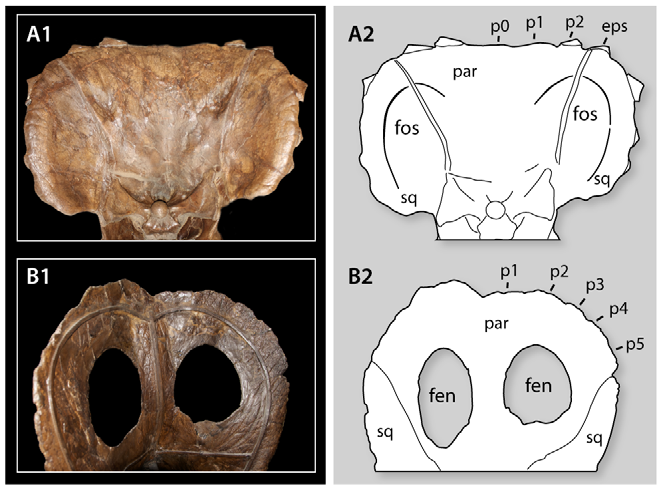
Comparison of frills in *Torosaurus* and *Triceratops*, showing the different position of the parietal fenestrae of *Torosaurus* and parietal fossae of *Triceratops*. A, *Triceratops* YPM 1823; B, *Torosaurus* ANSP 15192. Abbreviations: fen, fenestra; fos, parietal-squamosal fossa; par, parietal; sq, squamosal. P0, midline epiparietal; p1–p5 epiparietals 1–5; eps, epoccipital crossing the parietal-squamosal suture.

To assess the existence of putative incipient parietal fenestrae [31] in *Triceratops*, we provide new observations of the frill of *Triceratops*, focusing on the morphology of YPM 1823. In this specimen, the frill has a pair of prominent depressions on the ventral surface of the parietal, which are bordered by a raised platform of bone (Figure 9A). The depressions identified as incipient fenestrae in *Triceratops* are borne on the lateral margin of the parietal, and the depressions continue laterally onto the squamosals, and anteromedially onto the midline of the frill. These structures do not occupy the same position, nor do they have the same shape, as the openings in the frill of *Torosaurus* (Fig. 9B). In *Torosaurus*, the subcircular parietal fenestrae are entirely enclosed by the parietal, and are separated from the parietal-squamosal contact by a broad plate of bone that forms the lateral margin of the parietal; there is no fossa on the squamosal.

The skull of YPM 1823 is partially disarticulated, which allows the edge of the parietal to be viewed. Here it can be seen that the ventral fossae of the frill are formed by a moderately thick (∼10 mm thick at the edge) section of frill that is surrounded by a massive (∼30 mm thick) collar of bone around the caudal margin of the parietals and squamosals. By contrast, the frill in *Torosaurus* is relatively thin, typically up to 20 mm thick even in large individuals (e.g. YPM 1831) and at most 25 mm thick [31].

## Discussion

Our ontogenetic staging analysis shows that skulls assigned to *Torosaurus* are not consistently more mature than those assigned to *Triceratops*. Instead, both *Torosaurus* and *Triceratops* span a range of ontogenetic stages. Several *Torosaurus* specimens do appear to be near or at maturity, but others lack the full suite of features expected for a mature animal, including MOR 1122, YPM 1831, ANSP 15192, and USNM 15583 (Table S2). Furthermore, many *Triceratops* skulls exhibit extensive cranial fusion and a heavily rugose bone surface texture, suggesting that they are adults. The existence of small, mature individuals (Fig. 8) is striking, and has been interpreted by Scannella and Horner [31] as evidence that the timing of fusion is variable. We suggest instead that different animals may have stopped growing at different sizes, perhaps as a result of sexual dimorphism, with small adults representing females.

The fact that Scannella and Horner [31] found no mature *Triceratops* may simply reflect the fact that only two large *Triceratops* were sampled, and thus the limited sample may not have included the most mature individuals. Another possibility is that bone remodeling is simply not a reliable indicator of maturity. Although it seems intuitive that more heavily remodeled bone is older, this makes the assumption that the rate of remodeling is constant between individuals. However, experimental studies show that altering the loading regime of bones causes additional remodeling by secondary osteons [60], [61]; the rate of bone remodeling therefore changes as the loads experienced by the bones change. The degree of remodeling even varies within a single bone depending on the distribution of stresses [60] [62], which would mean that a paleontologist attempting to estimate the age of an animal could get different answers from different parts of the same fossil. This is not to say that bone remodeling is of no use in inferring age, but it does appear that further studies are required before we can conclude that it is.

Our study of the frill also casts doubt on the existence of intermediates linking *Torosaurus* and *Triceratops*. The depressions on the underside of the frill in *Triceratops* straddle the parietal and squamosal, while the openings in the frill in *Torosaurus* are located on the parietal. Furthermore, the ventral depressions in adult *Triceratops* are defined by a massive thickening of the posterior margin of the frill, which is absent in *Torosaurus*. Again, in this feature the frill of *Triceratops* does not preserve the intermediate morphology that would be expected if *Torosaurus* represented an adult *Triceratops*; for the *Triceratops* frill to transform into the *Torosaurus* frill, it would require 5–10 mm of bone be eroded from the entire posterior margin of the frill. The ventral depressions in *Triceratops* therefore appear to be a unique feature of the genus, and not a precursor of the parietal openings in *Torosaurus*.

Scannella and Horner have also proposed that USNM 2412 (“*Nedoceratops hatcheri*”) is transitional between the solid and fenestrate frill morphology [31], [41]; however, while USNM 2412 does possess an opening in the frill, the animal is clearly pathological, with large holes piercing not only the parietal but also the left and right squamosals [43]. The opening in the parietal is also irregular in shape, which strongly suggests that it is the result of injury or disease, and not a natural feature. The other side of the parietal is unfortunately damaged, which makes it difficult to resolve the issue. However, we would argue that there is simply no unambiguous evidence of intermediates between the solid and open frill morphologies, which is striking considering that so many *Triceratops* specimens are known. Another obstacle to interpreting USNM 2412 as a transitional form is that the animal appears to represent an old individual (Fig. 5), rather than an immature animal as would be predicted. Intermediates between *Triceratops* and *Torosaurus* are therefore unknown. While we concede that it is possible that such intermediates exist but simply have not been found, this seems unlikely given that so many horned dinosaur skulls are now known from the latest Cretaceous.

There are also other issues with synonymizing *Torosaurus* and *Triceratops*. The two differ in the number of epiparietals they possess [43]; *Torosaurus* has ten or more epiparietals [29], while *Triceratops* has between five and seven [31]. No specimens have ever been described with an intermediate number of epiparietals. Although it has been proposed that epiparietals were added late in life [31], it is unclear how epiparietals could be added, because these elements fuse to the frill in *Triceratops* without leaving space between them for the attachment of additional epiparietals [34], [35]. Scannella and Horner have more recently argued that the epiparietals split as the animals matured [41] but have not provided any examples of this process in the epiparietals.

The shape of the squamosals is another obstacle to synonymizing *Torosaurus* and *Triceratops*. *Torosaurus* squamosals are dorsally concave with a thickened inner margin; *Triceratops* squamosals are flat with a concave inner surface. Again, intermediates between the two have never been described. *Torosaurus* squamosals are also more elongate. Although Scannella and Horner have argued that *Torosaurus* and *Triceratops* squamosals share a common scaling relationship consistent with synonymy [31], this analysis is problematic. First, the reported correlation, with an R^2^ of 0.782, is exaggerated by autocorrelation. Plotting the squamosal length/width ratio versus against length means that squamosal length enters both terms in the regression, violating the assumption that the two terms are independent. Second, the two outliers in the regression are both *Torosaurus*: ANSP 15192 and YPM 1831 have squamosals that are relatively longer and narrower than expected by the regression. The fact that *Torosaurus* fits the model poorly is consistent with the hypothesis that more than one species is represented here.

Therefore, on the basis of multiple lines of evidence, the hypothesis of *Torosaurus*-*Triceratops* synonymy can be rejected. The results presented here have several implications for understanding the taxonomy of horned dinosaurs, and of dinosaurs in general. First, the developmental sequence confirms previous suggestions [52] that suture closure can be used to infer relative age in horned dinosaurs. Many chasmosaurine ceratopsids exhibit the same suite of cranial fusions found in *Torosaurus* and *Triceratops* [26], [57], [29], [58] and may have a similar sequence of fusion as well. Centrosaurine ceratopsids also show many of the fusions described here [32]. Thus, it should be possible to infer relative maturity of horned dinosaurs from a variety of species; this is significant because it would allow us to determine whether specimens are mature when diagnosing species. Our results also suggest, however, that adult body size is highly variable in ceratopsids, and so size cannot be used as a proxy for maturity, nor can it reliably be used to diagnose species.

Our study also shows that many features used to distinguish ceratopsid species appear before full maturity. Notably, parietal fenestration, epiparietal count, and squamosal shape are distinctive for *Torosaurus* and *Triceratops* regardless of their ontogenetic stage. It follows that immature dinosaurs are not necessarily nondiagnostic; some features are conserved over the course of development. Epoccipital arrangement is one such feature. Epoccipitals appear to represent osteoderms formed in association with scales. In extant diapsids, including lizards and alligators, scalation patterns are established in the embryo and subsequently scales may change in size, but not in number or arrangement [59], [60], [61]; scale patterns are therefore extremely useful for distinguishing living species. The inheritence of this developmental pattern by dinosaurs may explain why epoccipitals are so useful in ceratopsid taxonomy.

Finally, and more broadly, we argue that the approach used here provides a rigorous method for assessing synonymy in dinosaurs as it relates to ontogeny. Synonymy of fossil species is a major issue in paleontology. Dinosaurs are a particularly problematic case; as many as 50% of all named species have later been shown to be invalid [1], and a number of dinosaur genera have been shown to represent juveniles of previously described species. These include the tyrannosaur *Nanotyrannus* [37], the hadrosaur *Procheneosaurus* [63], the ceratopsid *Brachyceratops* [33], the pachycephalosaurs *Ornatotholus* [36], *Dracorex* [38], and perhaps *Homalocephale* [64] as well.

As a result, a more careful and rigorous approach to systematics is necessary. We agree with Scannella and Horner that it is necessary to determine whether variation between fossils can be explained by changes over the course of development before identifying a new species. In this study we provide a framework for testing the hypothesis of taxonomic synonymy resulting from ontogenetic change, by assessing support for three testable predictions: 1) overlap in stratigraphic and geographic occurrence, 2) consistent differences in relative maturity, and 3) the existence of morphological intermediates. Here, the application of this approach upholds the separation of *Triceratops* and *Torosaurus*. Although ontogenetic change represents a complicating factor for systematic paleontologists, the careful study of ontogeny in dinosaurs and other fossil animals will allow us to develop a robust systematic framework for better understanding the diversity of dinosaurs and other fossil animals.

## Supporting Information

Table S1 (DOCX)

Table S2 (DOCX)

Supporting Information S1 (DOCX)
