# Supplementary material for: Torosaurus Is Not Triceratops: Ontogeny in Chasmosaurine Ceratopsids as a Case Study in Dinosaur Taxonomy
Source: PLoS One. 2012 Feb 29;7(2):e32623. doi: 10.1371/journal.pone.0032623 (PMC3290593; doi:10.1371/journal.pone.0032623)
Supplement: Table S1 — (DOCX) [file pone.0032623.s001.docx]

**Table 1. Ontogenetic Characters.** For each character, ‘0’ represents the juvenile state, and ‘1’ and ‘2’ represent more mature states.

1. Postorbital horns, curvature: straight [0] curved posteriorly [1] curved forward [2]
2. Postorbital horns, length: horns short stubs [0] elongate [1]
3. Palpebral: separate from postorbital [0] or fused to postorbital [1]
4. Basioccipital: participates in foramen magnum [0] or excluded by exoccipitals [1]
5. Squamosal: short and narrow [0] or with long caudal blade and prominent anterolateral wing [1]
6. Parietals and squamosals: margins strongly scalloped [0] margins weakly scalloped or smooth [1]
7. Jugal: suborbital bar slender [0] or deep [1]
8. Postorbital horns, diameter: postorbital horns with narrow base [0] or base of horncore massive, expanded to cover the area posterodorsal to the orbit [1]
9. Postorbital horns, cornual sinuses: cornual sinuses weakly developed or absent [0] or cornual sinuses extensive and hollowing base of horncore [1]
10. Epoccipital ossifications with narrow bases [0] broad bases [1]
11. Nasals: narial bar depressed in lateral view, snout low [0] narial bar horizontal or upturned in lateral view, rostrum deep [1]
12. Parietosquamosal frill, texture: frill with smooth or striated texture [0] rugose texture and vascular grooves covering anterior part of frill [1] rugosity extends to caudal margin of frill [2]
13. Occipital condyle, fusion: exoccipitals and basioccipitals separate [0] or completely fused [1]
14. Frontals, fusion: frontals separate [0] or frontals fused along midline [1]
15. Epinasal, fusion: epinasal separate from nasals [0] or epinasal fused to nasals [1]
16. Nasals, fusion: nasals separate [0] or nasals fused along midline [1]
17. Postorbital and frontal: separate [0] or fused [1]
18. Lacrimal and prefrontal: separate [0] or fused [1]
19. Nasals, fusion: nasals separate from frontals, [0] or nasals fused to frontals [1]
20. Episquamosals: episquamosals separate [0] or episquamosals fused to squamosals [1]
21. Epiparietals: epiparietals separate [0] or epiparietals fused to parietal [1]
22. Epijugal, fusion: epijugal separate [0] or epijugal fused to jugal [1]
23. Rostral, fusion: rostral separate from premaxillae [0] or rostral fused to premaxillae [1]
24. Premaxillae, fusion: premaxillae separate from nasals [0] premaxillae fused to nasals [1]
