# Supplementary material for: Torosaurus Is Not Triceratops: Ontogeny in Chasmosaurine Ceratopsids as a Case Study in Dinosaur Taxonomy
Source: PLoS One. 2012 Feb 29;7(2):e32623. doi: 10.1371/journal.pone.0032623 (PMC3290593; doi:10.1371/journal.pone.0032623)
Supplement: Table S2 — (DOCX) [file pone.0032623.s002.docx]

**Table 2. Distribution of ontogenetically variable characters in *Triceratops* and *Torosaurus*.** “-“ = immature state, “X” =mature state 1, “XX” = mature state 2, blanks = missing data.

|  | Horn curvature | Horn elongation | Palpebral fusion | Exoccipitals contact | Long squamosal | Frill scalloping reduced | Jugal deep | Horn base massive | Sinuses invade horn | Epioccipitals broad | Rostrum deep | Fril surface rugose | Occipital condyle fused | Frontals fused | Epinasal fused to nasals | Nasals fused | Postorbital-frontal fusion | Lacrimal-prefrontal fusion | Nasal-frontal fusion | Episquamosals fused | Epiparietals fused | Epijugal fused | Rostral fused | Premaxilla-nasal fusion |
| --- | --- | --- | --- | --- | --- | --- | --- | --- | --- | --- | --- | --- | --- | --- | --- | --- | --- | --- | --- | --- | --- | --- | --- | --- |
|  | 1 | 2 | 3 | 4 | 5 | 6 | 7 | 8 | 9 | 10 | 11 | 12 | 13 | 14 | 15 | 16 | 17 | 18 | 19 | 20 | 21 | 22 | 23 | 24 |
| **UW 40634 Indet.** | - | - | - |  |  |  |  | - | - |  |  |  |  |  |  |  | - |  |  |  |  |  |  |  |
| **UCMP 154452 Triceratops** | - | - | - | - | - | - | - | - | - |  |  | - | - |  |  |  | - | - |  | - | - | - |  |  |
| **SMNH P2613.1 Indet.** | - | - | - |  |  |  |  | - | - |  |  |  |  |  |  |  |  |  |  |  |  |  |  |  |
| **AMNH 5006 Indet.** | X | - | - |  |  |  |  | - | - |  |  |  |  |  |  |  | - |  |  |  |  |  |  |  |
| **MOR 1199 *Triceratops*** | X | X | X | X | X | X | X | - | - | - | - | - | - | - | - | - | - | - | - | - | - | - | - | - |
| **MOR 1110 *Triceratops*** | X | X | X |  | X | X | X | X | X | X | - | - | - | - | - | - | - | - | - | - | - | - | - | - |
| **YPM 1821 *Triceratops*** | XX | X |  |  | X | X | X | X |  | X | X | X | X |  | - | - |  | - | - | - | - | - | - | - |
| **YPM 1823 *Triceratops*** | XX | X | X |  | X | X | X | X |  | X | X | XX | X | X | - | - | - | - | - | - | - | - | - | - |
| **MOR 1120 *Triceratops*** | XX | X | X |  | X | X | X | X | X | X | - | XX | X |  | X | X | X | - | - | - | - | - | - | - |
| **AMNH 5116 *Triceratops*** |  | X | X |  | X | X | X | X |  | X | X | X |  | X | X | X | X | - | - | - | - | - | - |  |
| **GMNH-PV 124 *Triceratops*** | XX | X | X |  | X | X |  | X |  |  |  | X | X | X | X | X | X | - | - | - | - | - | - | - |
| **UCMP 113697 *Triceratops*** | XX | X | X |  | X | X | X | X |  |  |  | - |  | X | X | X | X | - | - | - | - | - | - | - |
| **USNM 15583 *Torosaurus*** | XX | X | X |  | X | X | X | X |  |  |  |  |  |  |  |  |  | - |  | - |  | - |  |  |
| **YPM 1831 *Torosaurus*** | XX | X | X | X | X | X |  | X |  |  |  | X | - |  | X | X |  | X |  | - | - | - | - |  |
| **USNM 1201 *Triceratops*** | XX | X |  |  | X | X | X | X |  |  | X | X | X |  | X | X | X | X | - | - | - | - | - | - |
| **USNM 4928 *Triceratops*** | XX | X | X |  | X | X | X | X |  | X | X |  |  |  | X | X |  | X | X | - | - | - | - |  |
| **USNM 5740 *Triceratops* sp.** |  |  | X |  |  |  |  | X | X |  |  | X | X |  |  |  | X | X |  | X |  |  |  |  |
| **YPM 1830 *Torosaurus*** |  | X | X |  | X | X |  | X | X | X |  | XX | X |  | X | X | X | X | X | X |  |  |  |  |
| **YPM 1828 *Triceratops*** | XX | X | X |  | X | X |  | X |  | X | X | XX |  |  |  |  |  | X | X | X | X |  |  |  |
| **MNHN 1912.20 *Triceratops*** | XX | X | X |  | X | X | X | X |  | X | X | XX | X |  | X | X |  | X | X | X |  | X |  |  |
| **AMNH 970 *Triceratops*** |  |  | X |  | X | X |  | X |  | X |  | XX | X |  |  |  |  |  |  | X | X | X | - |  |
| **ANSP 15192 *Torosaurus*** | XX | X | X |  | X | X | X | X |  | X | X | XX | X | X | X | X |  | X | X | X | X | X | - | - |
| **YPM 1820 *Triceratops*** | XX | X | X |  |  |  | X | X | X |  | X | X | X | X | X | X | X | X | X |  |  | X | X | - |
| **MOR 1122 *Torosaurus*** | XX | X | X |  | X | X | X | X | X | X | X | X | X | X | X | X | X | X |  | X | X | X | X | - |
| **MPM VP6841 *Torosaurus*** |  |  |  |  | X | X | X |  |  | X |  | XX |  |  |  |  |  |  |  | X | X | X | X |  |
| **USNM 1205 *Triceratops*** |  |  | X |  | X | X | X | X | X | X | X | XX | X |  |  | X | X | X | X | X |  | X | X |  |
| **USNM 4741 *Triceratops*** |  |  |  |  |  |  |  |  |  |  |  | XX |  |  |  |  |  |  |  | X |  |  | X |  |
| **OMNH 10170 *Triceratops*** | XX | X |  |  | X | X |  | X | X | X |  | XX | X |  | X | X |  |  |  | X | X |  | X |  |
| **USNM 2100 *Triceratops*** | XX | X | X |  | X | X | X | X |  | X | X | XX | X | X | X | X | X | X | X | X | X | X |  | X |
| **USNM 2412 *Triceratops*** | XX | X | X |  | X | X | X | X |  | X | X | XX | X | X | X | X | X |  | X | X |  |  | X |  |
| **USNM 4720 *Triceratops*** | XX | X | X |  | X | X | X | X |  | X |  | XX | X | X | X | X | X | X | X | X |  | X |  | X |
| **YPM 1822 *Triceratops*** | XX | X | X | X | X | X | X | X |  | X | X | XX | X | X | X | X | X | X | X | X | X | X | X | X |
| **UCMP 128561 *Triceratops*** |  |  |  |  |  |  |  |  |  |  |  |  |  |  | X |  |  |  |  |  |  |  | X | X |
| **CM 1221 *Triceratops*** | XX | X | X |  | X | X | X | X |  | X | X | XX |  |  | X |  |  | X | X | X | X | X | X | X |
| **BSP 1964 I 458 *Triceratops*** | XX | X | X |  | X | X | X | X |  | X | X | XX | X | X | X | X | X |  | X | X | X | X | X | X |
| **BHI 6226 *Tatankaceratops*** | XX | X | X |  |  | X |  | - | - |  |  | XX | X |  | X | X | X | X |  | X | X |  | X | X |
