# Supplementary material for: Torosaurus Is Not Triceratops: Ontogeny in Chasmosaurine Ceratopsids as a Case Study in Dinosaur Taxonomy
Source: PLoS One. 2012 Feb 29;7(2):e32623. doi: 10.1371/journal.pone.0032623 (PMC3290593; doi:10.1371/journal.pone.0032623)
Supplement: Supporting Information S1 — (DOCX) [file pone.0032623.s003.docx]

**Online Supplementary Information For:**

Torosaurus *is not* Triceratops*: Ontogeny in Chasmosaurine Ceratopsids as a Case Study in Dinosaur Taxonomy*

**Contents:**

1. Character List
2. Character-Taxon Matrix
3. Material Studied
4. Results of Clustering Analyses
5. References

**1. Character List**

1. Basioccipital: participates in foramen magnum [0] or excluded by exoccipitals [1]
2. Postorbital horns, length: horns short stubs [0] elongate [1]
3. Postorbital horns, curvature: straight [0] curved posteriorly [1] curved forward [2]
4. Palpebral: separate from postorbital [0] or fused to postorbital [1]
5. Squamosal: short and narrow [0] or with long caudal blade and prominent anterolateral wing [1]
6. Parietals and squamosals: margins strongly scalloped [0] margins weakly scalloped or smooth [1]
7. Jugal: suborbital bar slender [0] or deep [1]
8. Postorbital horns, diameter: postorbital horns with narrow base [0] or base of horncore massive, expanded to cover the area posterodorsal to the orbit [1]
9. Postorbital horns, cornual sinuses: cornual sinuses weakly developed or absent [0] or cornual sinuses extensive and hollowing base of horncore [1]
10. Epoccipital ossifications with narrow bases [0] broad bases [1]
11. Nasals: narial bar depressed in lateral view, snout low [0] narial bar horizontal or upturned in lateral view, rostrum deep [1]
12. Parietosquamosal frill, texture: frill with smooth or striated texture [0] rugose texture and vascular grooves covering anterior part of frill [1] rugosity extends to caudal margin of frill [2]
13. Occipital condyle, fusion: exoccipitals and basioccipitals separate [0] or completely fused [1]
14. Frontals, fusion: frontals separate [0] or frontals fused along midline [1]
15. Epinasal, fusion: epinasal separate from nasals [0] or epinasal fused to nasals [1]
16. Nasals, fusion: nasals separate [0] or nasals fused along midline [1]
17. Postorbital and frontal: separate [0] or fused [1]
18. Lacrimal and prefrontal: separate [0] or fused [1]
19. Nasals, fusion: nasals separate from frontals, [0] or nasals fused to frontals [1]
20. Episquamosals: episquamosals separate [0] or episquamosals fused to squamosals [1]
21. Epiparietals: epiparietals separate [0] or epiparietals fused to parietal [1]
22. Epijugal, fusion: epijugal separate [0] or epijugal fused to jugal [1]
23. Rostral, fusion: rostral separate from premaxillae [0] or rostral fused to premaxillae [1]
24. Premaxillae, fusion: premaxillae separate from nasals [0] premaxillae fused to nasals [1]

**2. Character-Taxon Matrix**

MATRIX

UW_40634_Chasmosaurinae_indet. 000????00???????0???????

UCMP_154452_Triceratops_sp. 000000000??00???00?000??

SMNH_P2613.1_Chasmosaurinae_indet. 000????00???????????????

AMNH_5006_Chasmosaurinae_indet. 100????00???????0???????

MOR_1199_Triceratops_?horridus 111111100000000000000000

MOR_1110_Triceratops_prorsus 111?11111100000000000000

YPM_1821_Triceratops_prorsus_ 21??1111?1111?00?0000000

YPM_1823_Triceratops_prorsus_ 211?1111?112110000000000

MOR__1120_Triceratops_horridus_ 211?111111021?1110000000

AMNH_5116_Triceratops_horridus_ ?11?1111?111?1111000000?

GMNH-PV 124 Triceratops prorsus 211?11?1???1111110000000

UCMP_113697_Triceratops_prorsus 211?1111???0?11110000000

USNM_15583_Torosaurus_utahensis 211?1111?????????0?0?0??

YPM_1831_Torosaurus_latus 211111?1???10?11?1?0000?

USNM_1201_Triceratops_horridus_ 21??1111??111?1111000000

USNM_4928_Triceratops_horridus 211?1111?11???11?110000?

USNM_5740_Triceratops_sp. ??1????11??{1 2}1???11?1????

YPM_1830_Torosaurus_latus ?11?11?111?21?111111????

YPM_1828_Triceratops_horridus 211?11?1?112?????1111???

MNHN_1912.20_Triceratops_horridus 211?1111?1121?11?111?1??

AMNH_970_Triceratops_prorsus ??1?11?1?1?21??????1110?

ANSP_15192_Torosaurus_latus 211?1111?1121111?1111100

YPM_1820_Triceratops_horridus 211???111?1{1 2}1111111??110

MOR_1122_Torosaurus_latus 211?1111111{1 2}111111?11110

MPM_VP6841_Torosaurus_latus ????111??1?2???????1111?

USNM_1205_Triceratops_sp. ??1?111111121??11111?11?

USNM_4741_Triceratops_sp. ???????????2???????1??1?

OMNH_10170_Triceratops_prorsus 21??11?111?21?11???11?11

USNM_2100_Triceratops_horridus 211?1111?1121111111111?1

USNM_2412_Triceratops_horridus 211?1111?11211111?11??11

USNM_4720_Triceratops_horridus 211?1111?1?211111111?1?1

YPM_1822_Triceratops_prorsus 21111111?112111111111111

UCMP_128561_Triceratops_horridus ??????????????1???????11

CM_1221_Triceratops_prorsus 211?1111?112??1??1111111

BSP_1964_I_458_Triceratops_prorsus 211?1111?11211111?111111

BHI_6226_Tatankaceratops_sacrisonorum 211??1?00??21?1111?11?11

**3. Material Studied**

Specimens examined in the course of this study include ANSP 15192, AMNH 970, AMNH 5116, MPM VP6841, OMNH 10170, TMM 41480-1, UCMP 113697, USNM 1201, USNM 1205, USNM 2100, USNM 2412, USNM 4720, USNM 4741, USNM 5740, UW 40634, YPM 1820, YPM 1821, YPM 1822, YPM 1831, YPM 1823, YPM 1828, and YPM 1830.

Specimens coded from the literature include AMNH 5006 [[1](#_ENREF_1),[2](#_ENREF_2)], BHI_6226 [[3](#_ENREF_3)], BSP 1964 I 458/YPM 1834 [[4](#_ENREF_4)], CM 1221 [[5](#_ENREF_5),[6](#_ENREF_6)], MOR 1199, MOR 1110, and MOR 1120 [[7](#_ENREF_7),[8](#_ENREF_8)], MOR 1122, MNHN 1912.20 [[9](#_ENREF_9)], SMNH P2613.1 [[10](#_ENREF_10)], UCMP 128561 [[11](#_ENREF_11)], UCMP 154452 [[12](#_ENREF_12)], USNM 4928 [[13](#_ENREF_13)] and USNM 15583 [[14](#_ENREF_14)].

J. Scannella provided additional codings for MOR 1110, MOR 1120, and MOR 1199.

**4. Results of Clustering Analyses**

Analysis 1

Tree length = 33

Consistency index (CI) = 0.7879

Homoplasy index (HI) = 0.2121

CI excluding uninformative characters = 0.7500

HI excluding uninformative characters = 0.2500

Retention index (RI) = 0.9381

Rescaled consistency index (RC) = 0.7391/------------------------------------------------------------- UW 40634 Chasmos

|

+------------------------------------------------------------- UCMP 154452 Tric

|

+------------------------------------------------------------- SMNH P2613.1 Cha

|

| /--------------------------------------------------- AMNH 5006 Chasmo

| |

| | /----------------------------------------- MOR 1199 Tricera

| | |

| | | /------------------------------- MOR 1110 Tricera

\---------+ | |

| | | /-------------------- YPM 1821 Tricera

| | | |

| | | +-------------------- YPM 1823 Tricera

\---------+ | |

| | +-------------------- MOR 1120 Tricer

| | |

| | +-------------------- AMNH 5116 Tricer

| | |

| | +-------------------- GMNH-PV 124 Tric

\---------+ |

| +-------------------- UCMP 113697 Tric

| |

| +-------------------- USNM 15583 Toros

| |

| +-------------------- YPM 1831 Torosau

| |

| +-------------------- USNM 1201 Tricer

| |

| +-------------------- USNM 4928 Tricer

| |

\----------+ /---------- USNM 5740 Tricer

| |

| +---------- YPM 1830 Torosau

| |

| +---------- YPM 1828 Tricera

| |

| +---------- MNHN 1912.20 Tri

| |

| +---------- AMNH 970 Tricera

| |

| +---------- ANSP 15192 Toros

| |

| +---------- YPM 1820 Tricera

| |

| +---------- MOR 1122 Torosau

| |

| +---------- MPM VP6841 Toros

| |

| +---------- USNM 1205 Tricer

\---------+

+---------- USNM 4741 Tricer

|

+---------- OMNH 10170 Trice

|

+---------- USNM 2100 Tricer

|

+---------- USNM 2412 Tricer

|

+---------- USNM 4720 Tricer

|

+---------- YPM 1822 Tricera

|

+---------- UCMP 128561 Tric

|

+---------- CM 1221 Tricerat

|

+---------- BSP 1964 I 458 T

|

Strict consensus of 250000 trees: \--------- BHI 6226 Tatanka

/------------------------------------------------------------- UW 40634 Chasmos

|

+------------------------------------------------------------- UCMP 154452 Tric

|

+------------------------------------------------------------- SMNH P2613.1 Cha

|

| /------------------------------------------------------- AMNH 5006 Chasmo

| |

| | /------------------------------------------------- MOR 1199 Tricera

| | |

| | | /------------------------------------------- MOR 1110 Tricera

| | | |

\-----+ | | /------------------------------------- YPM 1821 Tricera

| | | |

| | | +------------------------------------- YPM 1823 Tricera

| | | |

| | | | /------------------------------- MOR 1120 Tricer

\-----+ | | |

| | | +------------------------------- AMNH 5116 Tricer

| | | |

| | | +------------------------------- GMNH-PV 124 Tric

| | | |

| | | +------------------------------- UCMP 113697 Tric

| | | |

| | | | /------ YPM 1831 Torosau

| | | +------------------------+

\-----+ +-----+ \------ USNM 1201 Tricer

| | |

| | | /------------------------ USNM 4928 Tricer

| | | |

| | | | /------------------ USNM 5740 Tricer

| | | | |

| | | | +------------------ YPM 1830 Torosau

| | | | |

| | | | +------------------ YPM 1828 Tricera

| | | | |

| | \------+ +------------------ MNHN 1912.20 Tri

| | | |

| | | +------------------ AMNH 970 Tricera

| | | |

| | | +------------------ ANSP 15192 Toros

\-----+ | |

| | | /------------ YPM 1820 Tricera

| \-----+ |

| | +------------ MOR 1122 Torosau

| | |

| | +------------ MPM VP6841 Toros

| | |

| | +------------ USNM 1205 Tricer

| | |

| | +------------ USNM 4741 Tricer

| | |

| | +------------ OMNH 10170 Trice

| | |

| | | /------ USNM 2100 Tricer

| \-----+ |

| | +------ USNM 4720 Tricer

| | |

| | +------ YPM 1822 Tricera

| | |

| +-----+------ UCMP 128561 Tric

| | |

| | +------ CM 1221 Tricerat

| | |

| | +------ BSP 1964 I 458 T

| | |

| | \------ BHI 6226 Tatanka

| |

| \------------ USNM 2412 Tricer

|

Adams Consensus \------------------------------------- USNM 15583 Toros

Analysis 2

MNHN 1912.20, USNM 5740, YPM 1828, YPM 1830, USNM 15583 (T. utahensis) deleted

Tree length = 33

Tree length = 33

Consistency index (CI) = 0.7879

Homoplasy index (HI) = 0.2121

CI excluding uninformative characters = 0.7500

HI excluding uninformative characters = 0.2500

Retention index (RI) = 0.9358

Rescaled consistency index (RC) = 0.7373

Strict consensus of 250000 trees:

/------------------------------------------------------------- UW 40634 Chasmos

|

+------------------------------------------------------------- UCMP 154452 Tric

|

+------------------------------------------------------------- SMNH P2613.1 Cha

|

| /----------------------------------------------------- AMNH 5006 Chasmo

| |

| | /---------------------------------------------- MOR 1199 Tricera

\-------+ |

| | /-------------------------------------- MOR 1110 Tricera

| | |

\------+ | /------------------------------- YPM 1821 Tricera

| | |

| | +------------------------------- YPM 1823 Tricera

\-------+ |

| | /----------------------- MOR 1120 Tricer

| | |

| | +----------------------- AMNH 5116 Tricer

| | |

\------+ +----------------------- GMNH-PV 124 Tric

| |

| +----------------------- UCMP 113697 Tric

| |

| +----------------------- YPM 1831 Torosau

| |

| +----------------------- USNM 1201 Tricer

| |

\-------+----------------------- USNM 4928 Tricer

|

| /--------------- AMNH 970 Tricera

| |

| +--------------- ANSP 15192 Toros

| |

| | /-------- YPM 1820 Tricera

| | |

| | +-------- MOR 1122 Torosau

| | |

| | +-------- MPM VP6841 Toros

\-------+ |

| +-------- USNM 1205 Tricer

| |

| +-------- USNM 4741 Tricer

| |

| +-------- OMNH 10170 Trice

| |

| +-------- USNM 2100 Tricer

\------+

+-------- USNM 2412 Tricer

|

+-------- USNM 4720 Tricer

|

+-------- YPM 1822 Tricera

|

+-------- UCMP 128561 Tric

|

+-------- CM 1221 Tricerat

|

+-------- BSP 1964 I 458 T

|

\-------- BHI 6226 Tatanka

Adams consensus of 250000 trees:

/------------------------------------------------------------- UW 40634 Chasmos

|

+------------------------------------------------------------- UCMP 154452 Tric

|

+------------------------------------------------------------- SMNH P2613.1 Cha

|

| /------------------------------------------------------- AMNH 5006 Chasmo

| |

| | /------------------------------------------------- MOR 1199 Tricera

\-----+ |

| | /------------------------------------------- MOR 1110 Tricera

| | |

\-----+ | /------------------------------------- YPM 1821 Tricera

| | |

| | +------------------------------------- YPM 1823 Tricera

\-----+ |

| | /------------------------------- MOR 1120 Tricer

| | |

| | +------------------------------- AMNH 5116 Tricer

\-----+ |

| +------------------------------- GMNH-PV 124 Tric

| |

| +------------------------------- UCMP 113697 Tric

| |

| | /------ YPM 1831 Torosau

\-----+------------------------+

| \------ USNM 1201 Tricer

|

| /------------------------ USNM 4928 Tricer

| |

| | /------------------ AMNH 970 Tricera

| | |

| | +------------------ ANSP 15192 Toros

| | |

\------+ | /------------ YPM 1820 Tricera

| | |

| | +------------ MOR 1122 Torosau

| | |

| | +------------ MPM VP6841 Toros

\-----+ |

| +------------ USNM 1205 Tricer

| |

| +------------ USNM 4741 Tricer

| |

| +------------ OMNH 10170 Trice

| |

| | /------ USNM 2100 Tricer

\-----+ |

| +------ USNM 4720 Tricer

| |

| +------ YPM 1822 Tricera

| |

+-----+------ UCMP 128561 Tric

| |

| +------ CM 1221 Tricerat

| |

| +------ BSP 1964 I 458 T

| |

| \------ BHI 6226 Tatanka

|

\------------ USNM 2412 Tricer

**5. References**

1. Brown B, Schlaikjer EM (1943) A study of the troödont dinosaurs with a description of a new genus and four new species. Bulletin of the American Museum of Natural History 82: 115-149.

2. Farke AA (2006) Morphology and ontogeny of the cornual sinuses in chasmosaurine dinosaurs (Ornithischia: Ceratopsidae). Journal of Paleontology 80: 780-785.

3. Ott CJ, Larson PL (2010) A new, small ceratopsian dinosaur from the latest Cretaceous Hell Creek Formation, northwest South Dakota, United States: a preliminary description. In: Ryan MJ, Chinnery BJ, Eberth DA, editors. New Perspectives on Horned Dinosaurs: The Royal Tyrrell Museum Ceratopsian Symposium. Bloomington: Indiana University Press. pp. 203-218.

4. Ostrom JH, Wellnhofer P (1986) The Munich specimen of *Triceratops* with a revision of the genus. Zitteliana 14: 111-158.

5. McIntosh JS (1981) Annotated catalogue of the dinosaurs (Reptilia, Archosauria) in the collections of Carnegie Museum of Natural History. Bulletin of the Carnegie Museum of Natural History 18: 1-67.

6. Forster CA (1996) Species resolution in *Triceratops*: cladistic and morphometric approaches. Journal of Vertebrate Paleontology 16: 259-270.

7. Horner JR, Goodwin MB (2006) Major cranial changes during *Triceratops* ontogeny. Proceedings of the Royal Society B 273: 2757-2761.

8. Horner JR, Goodwin MB (2008) Ontogeny of cranial epi-ossifications in *Triceratops*. Journal of Vertebrate Paleontology 28: 134-144.

9. Goussard F (2005) The skull of *Triceratops* in the palaeontology gallery, Muséum national d'Histoire naturelle, Paris. Geodiversitas 28: 467-476.

10. Tokaryk TT (1997) First evidence of juvenile ceratopsians (Reptilia: Ornithischia) from the Frenchman Formation (late Maastrichtian) of Saskatchewan. Canadian Journal of Earth Sciences 34: 1401-1404.

11. Cobabe E, Fastovsky DE (1987) *Ugrosaurus olsoni*, a new ceratopsian (Reptilia: Ornithischia) from the Hell Creek Formation of eastern Montana. Journal of Paleontology 61: 148-154.

12. Goodwin MB, Clemens WA, Horner JR, Padian K (2006) The smallest known *Triceratops* skull: new observations on ceratopsid cranial anatomy and ontogeny. Journal of Vertebrate Paleontology 26: 103-112.

13. Hatcher JB, Marsh OC, Lull RS (1907) The Ceratopsia. United States Geological Monograph 49: 1-300.

14. Sullivan RM, Boere AC, Lucas SG (2005) Redescription of the ceratopsid dinosaur *Torosaurus utahensis* (Gilmore, 1946) and a revision of the genus. Journal of Paleontology 79: 564-582.
